# Clinical obstacles to machine-learning POCUS adoption and system-wide AI implementation (The COMPASS-AI survey)

**DOI:** 10.1186/s13089-025-00436-2

**Published:** 2025-07-03

**Authors:** Adrian Wong, Nurul Liana Roslan, Rory McDonald, Julina Noor, Sam Hutchings, Pradeep D’Costa, Gabriele Via, Francesco Corradi

**Affiliations:** 1 Consultant Critical Care and Anaesthesia, King’s College Hospital, London, UK; 2 Department of Emergency Medicine, Hospital Kuala Lumpur, Kuala Lumpur, Malaysia; 3 Academic Department of Anaesthesia and Critical Care, Royal Centre for Defence Medicine, Birmingham, UK; 4 Dept of Emergency Medicine, Faculty of Medicine, Universiti Teknologi MARA, Kuala Lumpur, Malaysia; 5 Department of Critical Care, King’s College Hospital, King’s College Hospital NHS Foundation Trust, London, UK; 6 Sahyadri Hospital, Shastri Nagar branch, Pune, India; 7 Cardiac Anesthesia and Intensive Care, Ente Ospedaliero Cantonale (EOC), Istituto Cardiocentro Ticino, Università della Svizzera Italiana (USI), Lugano, Switzerland; 8 Department of Surgical, Medical, Molecular Pathology and Critical Care Medicine, University of Pisa, Pisa, Italy

**Keywords:** Point-of-care ultrasound (PoCUS), Artificial intelligence (AI), Machine learning (ML), Healthcare technology implementation

## Abstract

**Background:**

Point-of-care ultrasound (POCUS) has become indispensable in various medical specialties. The integration of artificial intelligence (AI) and machine learning (ML) holds significant promise to enhance POCUS capabilities further. However, a comprehensive understanding of healthcare professionals’ perspectives on this integration is lacking.

**Objective:**

This study aimed to investigate the global perceptions, familiarity, and adoption of AI in POCUS among healthcare professionals.

**Methods:**

An international, web-based survey was conducted among healthcare professionals involved in POCUS. The survey instrument included sections on demographics, familiarity with AI, perceived utility, barriers (technological, training, trust, workflow, legal/ethical), and overall perceptions regarding AI-assisted POCUS. The data was analysed by descriptive statistics, frequency distributions, and group comparisons (using chi-square/Fisher’s exact test and t-test/Mann-Whitney U test).

**Results:**

This study surveyed 1154 healthcare professionals on perceived barriers to implementing AI in point-of-care ultrasound. Despite general enthusiasm, with 81.1% of respondents expressing agreement or strong agreement, significant barriers were identified. The most frequently cited single greatest barriers were Training & Education (27.1%) and Clinical Validation & Evidence (17.5%). Analysis also revealed that perceptions of specific barriers vary significantly based on demographic factors, including region of practice, medical specialty, and years of healthcare experience.

**Conclusion:**

This novel global survey provides critical insights into the perceptions and adoption of AI in POCUS. Findings highlight considerable enthusiasm alongside crucial challenges, primarily concerning training, validation, guidelines, and support. Addressing these barriers is essential for the responsible and effective implementation of AI in POCUS.

**Graphical abstract:**

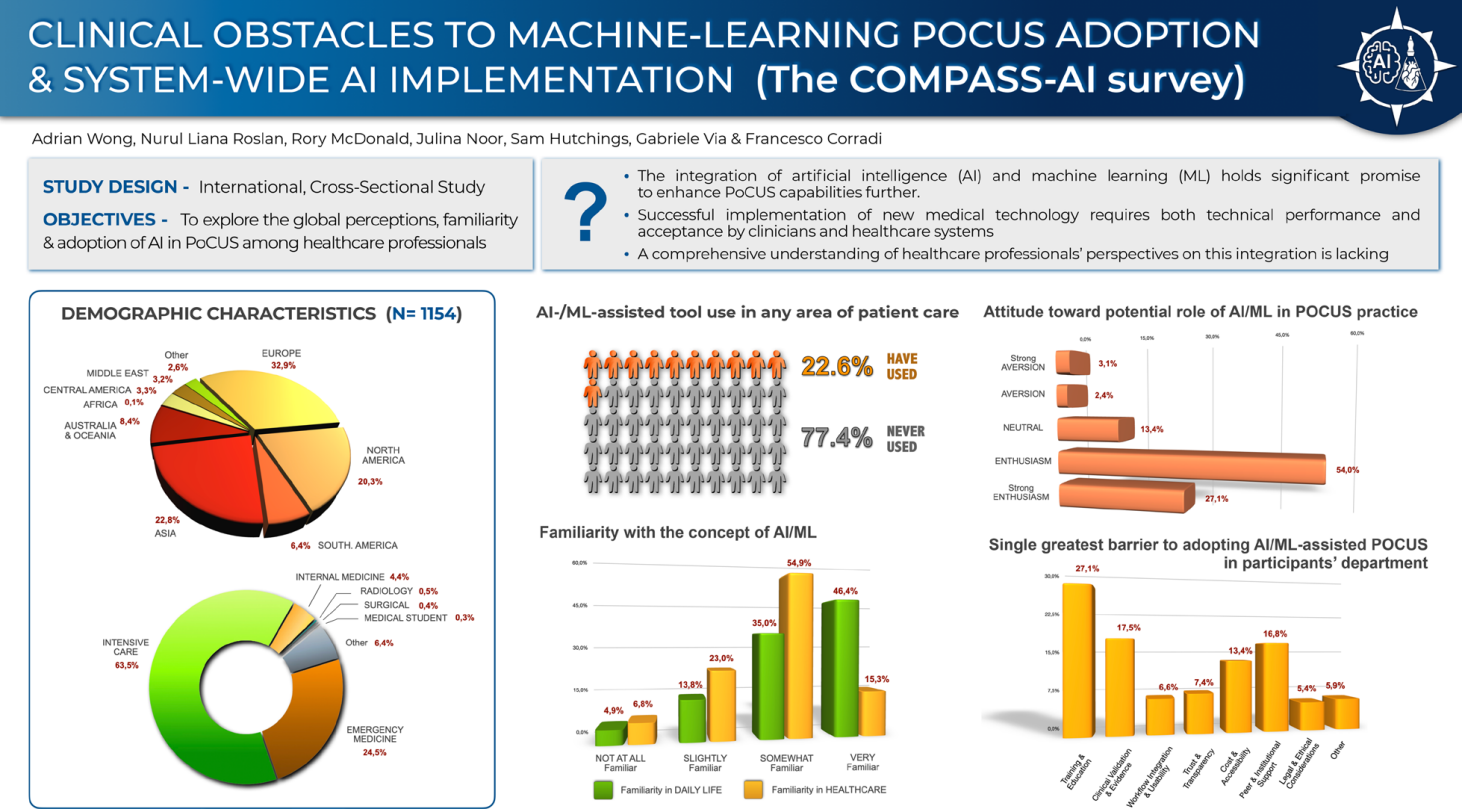

**Supplementary Information:**

The online version contains supplementary material available at 10.1186/s13089-025-00436-2.

## Introduction

Point-of-care ultrasound (POCUS) has become an indispensable tool across various clinical settings, including emergency medicine, critical care, and primary care. It facilitates timely bedside diagnosis and guides therapeutic decision-making. Concurrently, advancements in artificial intelligence (AI), particularly deep learning, coupled with increased computational power and large medical imaging datasets, have spurred interest in AI-driven algorithms to enhance the accuracy, consistency and efficiency of POCUS.

### Current state of AI in POCUS

The range of applications of AI in POCUS include automated image optimisation and acquisition, quality assessment, interpretation, and quantification (automatically measure physiological or anatomical parameters, such as cardiac output). Studies indicate AI can help identify anatomical structures [1]distinguish normal from abnormal scans [2] and detect specific pathologies e.g. cardiac wall-motion abnormalities [3, 4]pleural effusions [5]lung parenchymal diseases [6] and congestion [7, 8] with the potential to improve diagnostic accuracy.

AI-augmented tools show particular promise in guiding novice users [9–11] and reducing inter-operator variability [12]. Integrated AI software solutions could reduce barriers to training where supervision and resources are limited [8, 13]. By providing real-time guidance, automated feedback, and enhancing image quality and interpretation, AI-powered tools have the potential to flatten the learning curve for healthcare professionals, enabling quicker skill acquisition, boosting confidence, and expanding the accessibility of POCUS to a wider range of practitioners. However, like all branches of medicine, the use of AI into the medical education requires constructive engagement and requires addressing a multifaceted set of technical, educational, practical, and ethical considerations [14–16].

Despite these advancements, routine adoption of AI in POCUS has been limited. Known challenges include the need for robust prospective validation [17]user-friendly interfaces [18]regulatory approvals addressing safety and ethics [19]and substantial infrastructural requirements (data storage, algorithm updates, connectivity) [20]. Algorithm development has been complicated by variability in image quality, which is both operator and machine dependent [21]. Clinicians have expressed concerns about the “black box” nature of some AI models [22]potentially undermining trust. Furthermore, a lack of standardised guidelines training and certification translates to reluctance to use AI tools [14]alongside organisational barriers like workflow integration [23] and electronic medical record (EMR) compatibility [24, 25].

### Rationale for assessing barriers to adoption

Successful implementation of any new medical technology depends on both technical performance and acceptance by clinicians and healthcare systems. Understanding the reasons limiting the adoption of AI is crucial; factors such as the degree of clinician uncertainty regarding algorithm transparency, liability, and potential replacement of clinical judgment, along with institutional considerations of cost versus value, need to be evaluated.

### Aim and significance of this survey

The COMPASS-AI survey systematically assesses the perceived and actual barriers to adoption of AI across diverse clinical settings. By identifying critical obstacles, this study aims to inform strategies for improving training, regulatory frameworks, and technological integration, thereby facilitating a smoother transition towards AI-augmented POCUS.

## Methodology

### Study design and setting

An international, cross-sectional survey design was used to investigate barriers to adoption of AI in POCUS. The survey instrument, based on literature reviews and pilot testing for clarity and relevance, covered domains including demographics, clinical practice, AI utility perception, training, resources, trust, and workflow challenges. The study protocol received approval from the King’s College London Institutional Review Board (IRB registration number 46603), and participants provided digital informed consent.

### Participant recruitment

To capture a broad, international perspective, healthcare professionals engaged in POCUS were recruited through a multi-channel strategy. This involved dissemination via the membership lists of multiple international professional societies and targeted promotion on professional social media networks. The survey was disseminated using a unique REDCap link, with two reminders sent to encourage participation. Due to this wide-reaching, network-based dissemination strategy, a precise denominator could not be calculated, precluding the reporting of a formal response rate.

### Survey instrument

The survey instrument was developed through a multi-step process. Initial domains were identified from a review of literature on barriers to AI and technology adoption in healthcare [13, 15, 17]. The structure was designed to align with established implementation science frameworks, ensuring a comprehensive assessment of factors known to influence technology acceptance. The themes covered (utility, training, resources, trust, workflow) correspond well with core constructs of models like the Unified Theory of Acceptance and Use of Technology (UTAUT), such as Performance Expectancy, Effort Expectancy, and Facilitating Conditions [26]. The draft instrument underwent pilot testing for clarity and relevance and was refined by the author group, comprising international experts in POCUS. (see appendix).

### Statistical analysis

Data was exported from REDCap for analysis. Descriptive statistics (means, medians, frequencies, percentages, IQR) were calculated. Chi-square or Fisher’s exact tests were used for categorical variable comparisons, and independent-samples t-tests or Mann-Whitney U tests for continuous/ordinal variables based on data distribution. Group comparisons were performed based on region, specialty, and experience level for key perception questions.

## Results

### Section A: respondents’ demographics

#### Respondents’ demographics

A total of 1154 healthcare professionals participated in the survey, and all responses were included in the analysis. Respondents were predominantly from Europe (*n* = 380, 32.9%), Asia (*n* = 263, 22.8%), and North America (*n* = 234, 20.3%). The most represented medical specialties were Intensive care (*n* = 730, 63.5%) and Emergency medicine (*n* = 281, 24.5%). The cohort was largely experienced, with the majority having worked in healthcare for more than 10 years (*n* = 612, 53.6%) or 6–10 years (*n* = 337, 29.5%). Reflecting their expertise, a high frequency of POCUS usage was reported, with most participants using or interpreting POCUS multiple times per day (*n* = 603, 52.4%) or several times per week (*n* = 448, 38.9%).

#### Familiarity with AI

Regarding familiarity with AI, respondents showed varied exposure. While many were very familiar (*n* = 535, 46.4%) or somewhat familiar (*n* = 404, 35.0%) with AI in daily life, familiarity specifically within healthcare was slightly lower, with the largest group being somewhat familiar (*n* = 633, 54.9%).

A strong positive correlation was observed between familiarity in daily life and familiarity in healthcare (Spearman’s rho = 0.585, *p* < 0.001).

A majority of the respondents (*n* = 891, 77.4%) indicated they had not previously used an AI- or ML-assisted tool in patient care.

#### Perceptions of AI-assisted POCUS

Respondents’ perceptions regarding the utility and integration of AI in POCUS were explored across several domains.

### Section B: perceived utility and clinical integration

Analysis of responses concerning the perceived utility and clinical integration of AI-assisted POCUS indicates a generally positive outlook on its potential benefits. For instance, a significant majority of respondents agreed (*n* = 582, 54.2%) or strongly agreed (*n* = 212, 19.7%) that AI could improve the speed of diagnosis in the ICU. Similarly, the potential to reduce inter-operator variability in ultrasound interpretation garnered high agreement (Agree: *n* = 610, 56.7%; Strongly Agree: *n* = 345, 32.1%). Many also believed AI would help improve the accuracy of their ultrasound interpretations (Agree: *n* = 495, 46.0%; Strongly Agree: *n* = 197, 18.3%) and increase confidence in clinical decisions if access to AI-assisted interpretations was available (Agree: *n* = 453, 42.1%; Strongly Agree: *n* = 245, 22.8%). When asked if they believed AI could be integrated seamlessly into their current POCUS workflow, perceptions were varied. A considerable portion agreeing (*n* = 475, 44.2%) or strongly agreeing (*n* = 177, 16.5%), while a notable number were neutral (*n* = 271, 25.2%) or disagreed (Disagree: *n* = 134, 12.5%; Strongly Disagree: *n* = 17, 1.6%).

### Section C: technological and training barriers

Responses related to technological and training barriers highlight significant concerns in these areas. A substantial majority of participants felt they lacked sufficient training to effectively use AI-assisted ultrasound tools (Disagree: *n* = 506, 48.1%; Strongly Disagree: *n* = 98, 9.3%), and a high percentage also perceived available training resources as inadequate (Disagree: *n* = 473, 44.9%; Strongly Disagree: *n* = 96, 9.1%). A lack of standardized training or credentialing for AI in POCUS was widely considered a significant barrier, with many agreeing (*n* = 471, 44.7%) or strongly agreeing (*n* = 407, 38.7%). Insufficient local expertise or technical support was also identified as a substantial impediment to adoption by a large proportion of respondents (Agree: *n* = 470, 44.7%; Strongly Agree: *n* = 304, 28.9%).

### Section D: trust, accuracy, and reliability concerns

Analysis of the responses concerning trust, accuracy, and reliability reveals nuanced perspectives. While a majority expressed some level of trust in AI algorithms for accurate interpretations (Agree: n = 503, 48.8%; Strongly Agree: n = 212, 20.6%), a considerable number articulated concerns that AI errors could lead to incorrect diagnoses or treatments (Agree: n = 457, 44.4%; Strongly Agree: n = 100, 9.7%). The necessity of verifying every AI-generated finding with personal interpretation before making a clinical decision was strongly emphasized by a large proportion of respondents (Agree: n = 511, 49.6%; Strongly Agree: n = 249, 24.2%). The ‘black box’ nature (lack of explainability) of AI outputs was perceived by many as a factor that reduces trust (Agree: *n* = 347, 33.7%; Strongly Agree: *n* = 85, 8.3%; Neutral: *n* = 455, 44.2%). Conversely, regulatory approval and strong evidence validating AI tools were widely seen as factors that would significantly increase the willingness to use these technologies (Agree: *n* = 473, 45.9%; Strongly Agree: *n* = 417, 40.5%).

### Section E: workflow and resource barriers

Perceptions regarding workflow and resource implications demonstrate divided opinions. While a considerable portion of respondents disagreed (*n* = 441, 43.0%) or strongly disagreed (*n* = 59, 5.8%) that implementing AI would slow down their workflow, a notable number remained neutral (*n* = 365, 35.6%) or felt it would (Agree: *n* = 137, 13.4%; Strongly Agree: *n* = 23, 2.2%). The cost of acquiring and maintaining AI-enabled ultrasound machines was perceived as prohibitive by a substantial percentage of the cohort (Agree: *n* = 173, 16.9%; Strongly Agree: *n* = 88, 8.6%; Neutral: *n* = 441, 43.0%). Opinions were mixed regarding the sufficiency and complexity of integrating AI outputs into electronic health records (Agree: *n* = 147, 14.3%; Strongly Agree: *n* = 44, 4.3%; Neutral: *n* = 423, 41.3%; Disagree: *n* = 386, 37.7%; Strongly Disagree: *n* = 25, 2.4%). The requirement for additional technical support or staff to effectively utilize AI was acknowledged by many (Agree: *n* = 299, 29.2%; Strongly Agree: *n* = 68, 6.6%; Neutral: *n* = 368, 35.9%).

### Section F: legal, ethical, and cultural barriers

Responses pertaining to legal, ethical, and cultural barriers indicate that these factors are also relevant. Concerns about liability if AI-assisted interpretations are incorrect were present among a notable portion of respondents (Agree: *n* = 241, 24.0%; Strongly Agree: *n* = 47, 4.7%; Neutral: *n* = 399, 39.8%). While opinions on data privacy and security concerns were more varied, 154 respondents (15.4%) agreed and 48 (4.8%) strongly agreed that these are significant barriers, while 410 (40.9%) disagreed and 100 (10.0%) strongly disagreed. A lack of clear institutional or professional guidelines on AI use in POCUS was a major factor contributing to hesitation in adoption (Agree: *n* = 369, 36.8%; Strongly Agree: *n* = 438, 43.7%). Cultural resistance to new technology among colleagues or leadership was also identified as an inhibiting factor by a considerable number of participants (Agree: *n* = 319, 31.8%; Strongly Agree: *n* = 120, 12.0%; Neutral: *n* = 331, 33.0%). Conversely, official endorsements or recommendations by professional societies were widely believed to increase the willingness to adopt AI-assisted POCUS (Agree: *n* = 375, 37.4%; Strongly Agree: *n* = 214, 21.4%; Neutral: *n* = 276, 27.5%).

### Overall opinions

When asked to identify the single greatest barrier to adopting AI-assisted POCUS, the most frequently cited factor was Training & Education (*n* = 271, 27.1%), followed by Clinical Validation & Evidence (*n* = 175, 17.5%), and Peer & Institutional Support (*n* = 168, 16.8%).

Overall, despite the acknowledged barriers, a strong majority of respondents expressed enthusiasm about the potential role of AI in improving POCUS practice (Agree: *n* = 542, 54.0%; Strongly Agree: *n* = 272, 27.1%).

### Group comparisons

Group comparisons revealed statistically significant differences in opinions regarding several perceived barriers to the implementation of AI in POCUS across different demographic groups.

Analysis by region demonstrated significant differences regarding the perceived potential for AI to reduce inter-operator variability (H = 41.929, *p* < 0.001), the perception of a lack of standardized training or credentialing as a significant barrier (H = 64.178, *p* < 0.001), and the importance of regulatory approval and strong evidence validating AI tools (H = 54.221, *p* < 0.001). While a high percentage across all regions agreed that a lack of standardized training is a barrier, respondents from Australia/Oceania and North America reported particularly high levels of agreement. Similarly, agreement on the potential to reduce inter-operator variability was high across regions, with Australia/Oceania showing 100% agreement (Agree + Strongly Agree). The importance of regulatory approval and evidence was also widely agreed upon, again with high agreement in Australia/Oceania and the Middle East.

Analysis by specialty also showed significant differences regarding the perceived potential for AI to reduce inter-operator variability (H = 23.449, *p* = 0.001), the importance of regulatory approval and strong evidence validating AI tools (H = 18.088, *p* = 0.006), the potential increase in confidence by using AI tools (H = 38.450, *p* < 0.001), and the perception of a lack of standardized training or credentialing as a significant barrier (H = 42.112, *p* < 0.001). While Intensive Care respondents generally reported higher agreement scores on many aspects, there were variations. For instance, Intensive Care had a high percentage agreeing that lack of standardized training is a barrier (87.7%), while Emergency Medicine also showed a high percentage (77.1%). Regarding increased confidence with AI tools, Intensive Care reported a higher percentage of agreement (71.3%) compared to Emergency Medicine (49.6%).

Comparisons based on duration of clinical experience indicated significant differences for the perceived lack of standardized training or credentialing as a barrier (H = 53.929, *p* < 0.001), the importance of regulatory approval and strong evidence validating AI tools (H = 45.552, *p* < 0.001), and the expected confidence gain from AI integration (H = 113.939, *p* < 0.001). Clinicians with 1–5 years of experience reported the highest percentage of agreement regarding expected confidence gain from AI integration (86.1%), while the most experienced group (> 10 years) reported a lower percentage (52.5%). Those respondents with 1–10 years of experience showed particularly high levels of agreement that a lack of standardized training is a significant barrier (6–10 years: 88.5%, 1–5 years: 85.4%). The importance of regulatory approval and evidence also garnered high agreement across experience levels, with 1–5 years showing the highest percentage of agreement (93.9%).

## Discussion

This global survey provides valuable insights into the perceptions and anticipated challenges surrounding the integration of AI in POCUS among a large cohort of healthcare professionals.

There were notable differences in opinions that varied with geographical location, specialty, seniority etc. It was not too surprising to find that younger colleagues and colleagues in developing nations appear to embrace new technology more readily, given the likely exposure to smart gadgets in their personal lives.

Our findings confirm considerable enthusiasm and recognition of the significant potential for AI to enhance POCUS practice, particularly in areas like image optimization, interpretation assistance, and potentially reducing inter-operator variability.

However, the survey also underscores critical concerns and highlights that the successful adoption of AI is far from solely a technological challenge. The most frequently cited barriers – training and education, clinical validation and evidence, and peer and institutional support – point towards systemic and human factors that require focused attention. Addressing these challenges represents a significant opportunity to pave the way for successful implementation.

It is vital to contextualize the role of AI within the inherently dynamic nature of POCUS. AI algorithms, regardless of sophistication, can only process the information present in the patient at a specific moment. They cannot predict or account for the rapid, unpredictable changes in clinical trajectory that frequently occur in high-acuity settings like the OR, ICU, or ED due to external factors. Therefore, a negative AI-assisted finding at a single point in time necessitates ongoing clinical vigilance.

Furthermore, the performance of any AI tool is fundamentally linked to the quality of the input data – “garbage in, garbage out.” Poor quality ultrasound images will limit AI performance; in these situations, AI is able to actively assist in improving image quality, thereby improving the basis for subsequent analysis, a potential benefit suggested by our findings [11].

Critically, AI-enhanced POCUS must always be viewed as an adjunct, a powerful tool to augment, not replace, clinical expertise [18]. Its output necessitates careful interpretation within the full clinical context and must be followed by an appropriate action plan. Diagnosis or prediction facilitated by AI-POCUS, such as identifying early signs of AKI [27]offers an opportunity for earlier intervention, although it does not equate to prevention; clinical action remains paramount.

AI holds significant promise as a personalized, accessible instructor, potentially accelerating learning and helping to address the shortage of POCUS instructors [28, 29]. However, as POCUS is more than just image acquisition, it requires integration into the clinical context, this highlights the need to enhance educational strategies, both for learners as well as for the POCUS instructors who will guide the next generation in using these augmented tools responsibly.

Looking ahead, the most significant advantages and exciting opportunities for AI in POCUS may lie in its capacity to integrate information from multiple sources – ultrasound images combined with ECG signals, EEG, or ventilator waveforms – offering a more holistic view of the patient. Furthermore, AI’s ability to perform real-time, multiframe analysis can potentially detect subtle patterns invisible to the human eye [30]such as differentiating complex pleural line morphologies [31–33]unlocking new diagnostic capabilities.

Finally, building trust and ensuring responsible adoption hinges on robust, independent validation. Our survey respondents overwhelmingly agreed on the importance of regulatory approval and strong evidence. This must include external validation of AI software by clinicians, moving beyond industry-sponsored studies, to ensure reliability and clinical applicability, as highlighted by concerns stemming from experiences with other hemodynamic monitoring tools. Achieving this validation is key to unlocking the full potential and widespread adoption of AI in POCUS.

### Comparison with existing literature

While prior studies focused on specific AI applications or single centres, this study provides a unique global perspective. The enthusiasm we have found aligns with literature on AI’s potential in medical imaging [34–37]. Our findings expand on this by comprehensively assessing perceptions, familiarity, adoption patterns, and specific barriers like the need for XAI (Explainable AI) to address “black box” concerns, and the critical role of training and guidelines.

### Implications for practice

1. **Training Programs**: Develop comprehensive, hands-on training programs addressing specific skills for using AI-POCUS tools competently.
2. **Building Trust**: Enhance transparency and explainability of AI algorithms and provide robust validation evidence through regulatory approval.
3. **Addressing Implementation Challenges**: Develop strategies for cost-effectiveness, standardized integration protocols, clear guidelines, and adequate local technical/peer support.
4. **Tailored Strategies**: Acknowledge and address the differing needs and perspectives based on user geography, specialty, and experience.

### Limitations of our study

Potential response bias exists, possibly favouring those interested in AI. Data relies on self-reporting, subject to recall bias. The cross-sectional design only offers a snapshot; longitudinal studies are needed to track evolving perceptions. The analysis report noted data mapping issues for some questions (e.g., ‘Available training resources’, ‘Overall enthusiasm’), which limited analysis on those specific items.

### Future research directions

Longitudinal studies are needed to track adoption and perception changes. Qualitative methods (interviews, focus groups) could offer deeper insights into user experiences. Rigorous clinical trials are essential to evaluate the clinical effectiveness and safety of specific AI tools.

## Conclusion

In conclusion, while the integration of AI in POCUS presents challenges that require deliberate attention, our global survey reveals a strong undercurrent of enthusiasm and a clear recognition of its transformative potential. Addressing the critical needs for comprehensive training (for both users and instructors), ensuring rigorous independent validation and explainability to build trust, developing clear guidelines, and providing adequate support are essential steps. Embracing these challenges as opportunities for innovation and collaboration among clinicians, researchers, industry developers, and policymakers, grounded in a realistic understanding of AI’s capabilities and limitations, is crucial for the responsible and effective adoption of AI in POCUS, ultimately aiming to significantly improve patient care and outcomes.

## Electronic supplementary material

Below is the link to the electronic supplementary material.

Supplementary Material 1

Supplementary Material 2

## Data Availability

Survey tool and raw datasheet available upon requests.
