# Supplementary material for: Clinical obstacles to machine-learning POCUS adoption and system-wide AI implementation (The COMPASS-AI survey)
Source: Ultrasound J. 2025 Jul 3;17:32. doi: 10.1186/s13089-025-00436-2 (PMC12229359; doi:10.1186/s13089-025-00436-2)
Supplement: Supplementary file 1 — Supplementary Material 1 [file 13089_2025_436_MOESM1_ESM.pdf]

CLINICAL OBSTACLES TO MACHINE-LEARNING POCUS ADOPTION & SYSTEM-WIDE AI IMPLEMENTATION (The COMPASS-AI survey)

Section A: Demographics and Background

Clinical Obstacles to Machine-learning POCUS Adoption and System-wide AI Implementation (COMPASS-AI) Survey
Exploring Barriers to AI/ML Adoption in ICU POCUS

Which region do you practise in?	◯ Europe<br>◯ North America<br>◯ Central America<br>◯ South America<br>◯ Asia<br>◯ Middle East<br>◯ Australia/Oceania<br>◯ Other (please specify)
Other (please specify)	__________________________________
Which is your main specialty?	◯ Intensive care<br>◯ Emergency medicince<br>◯ Internal medicine<br>◯ Surgical<br>◯ Radiology<br>◯ Medical Student<br>◯ Other
Other, please specify	__________________________________
What type of hospital do you work in?	◯ University teaching hospital<br>◯ University affiliated hospital<br>◯ General hospital<br>◯ Other
Other, please specify	__________________________________

What is your primary role in the department?	◯ Attending Physician (Consultant)<br>◯ Fellow / Resident / Trainee<br>◯ Nurse<br>◯ Physician Assistant / Nurse Practitioner<br>◯ Medical Student<br>◯ Administrator / Manager<br>◯ Other, please specify
Other, please specify:	________________________________
How many years have you worked in healthcare?	◯ Less than 1 year<br>◯ 1-5 years<br>◯ 6-10 years<br>◯ More than 10 years
How frequently do you use or interpret POCUS?	◯ Multiple times per day<br>◯ Several times per week<br>◯ About once per week<br>◯ Less than once per week<br>◯ Never
How familiar are you with the concept of AI/ML in your daily life?	◯ Very familiar (routinely use AI tools e.g. ChatGPT, GEMINI, Siri)<br>◯ Somewhat familiar (have read about it, use it occasionally or seen demonstrations)<br>◯ Slightly familiar (have heard about it, but minimal exposure)<br>◯ Not at all familiar
How familiar are you with the concept of AI/ML in healthcare (not necessarily in POCUS)?	◯ Very familiar (routinely use or study AI in some aspect of practice)<br>◯ Somewhat familiar (have read about it or seen demonstrations)<br>◯ Slightly familiar (have heard about it, but minimal exposure)<br>◯ Not at all familiar
Have you ever used an AI- or ML-assisted tool in any area of patient care?	◯ Yes<br>◯ No
Please specify the type of AI/ML tool you have used:	________________________________

Section B: Perceived Utility and Clinical Integration

Please indicate your level of agreement with the following statements.

	Strongly disagree	Disagree	Neutral	Agree	Strongly agree
AI/ML-assisted POCUS could improve the speed of diagnosis in the ICU.	○	○	○	○	○
AI/ML-assisted POCUS would help improve the accuracy of my ultrasound interpretations.	○	○	○	○	○
Integrating AI/ML into my current POCUS workflow would be relatively seamless.	○	○	○	○	○
AI/ML-assisted POCUS could reduce inter-operator variability in ultrasound interpretation.	○	○	○	○	○
I would feel more confident in my clinical decisions if I had access to AI/ML-assisted interpretations.	○	○	○	○	○

Section C: Technological and Training Barriers

Please indicate your level of agreement with the following statements.

	Strongly disagree	Disagree	Neutral	Agree	Strongly agree
I have sufficient training to use AI/ML-assisted ultrasound tools effectively.	○	○	○	○	○
Available training resources (e.g., workshops, online modules) are adequate to learn AI/ML-enhanced POCUS.	○	○	○	○	○
A lack of standardized training or credentialing for AI/ML in POCUS is a significant barrier.	○	○	○	○	○
Insufficient local expertise or technical support hinders the adoption of AI/ML-assisted POCUS.	○	○	○	○	○

Section D: Trust, Accuracy, and Reliability Concerns

Please indicate your level of agreement with the following statements.

	Strongly disagree	Disagree	Neutral	Agree	Strongly agree
I trust AI/ML algorithms to provide accurate ultrasound interpretations.	○	○	○	○	○
I am concerned that AI/ML errors could lead to incorrect diagnoses or treatments.	○	○	○	○	○
I would want to verify every AI/ML-generated finding with my own interpretation before making a clinical decision.	○	○	○	○	○
The "black box" nature (lack of explainability) of AI/ML outputs reduces my trust in them.	○	○	○	○	○
Regulatory approval and strong evidence validating AI/ML tools would increase my willingness to use them	○	○	○	○	○

Section E: Workflow and Resource Barriers

Please indicate your level of agreement with the following statements.

	Strongly disagree	Disagree	Neutral	Agree	Strongly agree
Implementing AI/ML in POCUS would slow down my workflow.	○	○	○	○	○
The cost of acquiring and maintaining AI/ML-enabled ultrasound machines is prohibitive.	○	○	○	○	○
Integration of AI/ML outputs into the health records (patient notes) is insufficient or too complex.	○	○	○	○	○
Additional technical support or staff would be required to effectively utilize AI/ML in POCUS.	○	○	○	○	○

Section F: Legal, Ethical, and Cultural Barriers

Please indicate your level of agreement with the following statements.

	Strongly disagree	Disagree	Neutral	Agree	Strongly agree
Concerns about liability if AI/ML-assisted interpretations are incorrect discourage me from using these tools.	○	○	○	○	○
Data privacy and security concerns about patient information used to train AI systems are significant barriers	○	○	○	○	○
A lack of clear institutional or professional guidelines on AI/ML use in POCUS makes me hesitant to adopt it.	○	○	○	○	○
Cultural resistance to new technology among colleagues or leadership in my ICU inhibits the introduction of AI/ML tools.	○	○	○	○	○
Official endorsements or recommendations by professional societies would make me more willing to adopt AI/ML-assisted POCUS.	○	○	○	○	○

Section G: Overall Perceptions and Open-Ended Feedback

Overall, I am enthusiastic about the potential role of AI/ML in improving POCUS practice.

- ◯ Strongly disagree
- ◯ Disagree
- ◯ Neutral
- ◯ Agree
- ◯ Strongly agree

In your opinion, what is the single greatest barrier to adopting AI/ML-assisted POCUS in your department?

- ◯ Training & Education
- ◯ Clinical Validation & Evidence
- ◯ Workflow Integration & Usability
- ◯ Trust & Transparency
- ◯ Cost & Accessibility
- ◯ Peer & Institutional Support
- ◯ Legal & Ethical Considerations
- ◯ Other

Other (please specify)

____

Do you have any additional comments or suggestions regarding AI/ML integration into POCUS in healthcare?

____

If you would like to be kept updated on the results of the survey or be open to possible future collaborations, please leave your email here.

____
