# Supplementary material for: Clinical obstacles to machine-learning POCUS adoption and system-wide AI implementation (The COMPASS-AI survey)
Source: Ultrasound J. 2025 Jul 3;17:32. doi: 10.1186/s13089-025-00436-2 (PMC12229359; doi:10.1186/s13089-025-00436-2)
Supplement: Supplementary file 2 — Supplementary Material 2 [file 13089_2025_436_MOESM2_ESM.pdf]

CLINICAL OBSTACLES TO MACHINE-LEARNING POCUS ADOPTION & SYSTEM-WIDE AI IMPLEMENTATION (The COMPASS-AI survey)

Which region do you practise in?		
EUROPE	380	**32,9%**
NORTH AMERICA	234	**20,3%**
SOUTH AMERICA	74	**6,4%**
ASIA	263	**22,8%**
AUSTRALIA/OCEANIA	97	**8,4%**
AFRICA	1	**0,1%**
CENTRAL AMERICA	38	**3,3%**
MIDDLE EAST	37	**3,2%**
Other	30	**2,6%**
**Total**	**1154**	**100,0%**

Which is your main specialty?		
Emergency medicince	281	**24,5%**
Intensive care	730	**63,5%**
Internal medicine	50	**4,4%**
Radiology	6	**0,5%**
Surgical	5	**0,4%**
Medical student	4	**0,3%**
Other	73	**6,4%**
	**1149**	**100,0%**

What type of hospital do you work in?	
University teaching hospital	572
General hospital	462
University affiliated hospital	61
Other	55
**Total**	**1150**

What is your primary role in the department?	Other, please specify:	
Attending Physician (Consultant)	548	**48,4%**
Fellow / Resident / Trainee	544	**48,0%**
Nurse	8	**0,7%**
Administrator / Manager	12	**1,1%**
Other, please specify	21	**1,9%**
**Total**	**1133**	**100,0%**

How many years have you worked in healthcare?		
1-5 years	193	**16,9%**
6-10 years	337	**29,5%**
More than 10 years	612	**53,6%**
**Total**	**1142**	**100,0%**

How frequently do you use or interpret POCUS?		
Never	18	**1,6%**
Less than once per week	43	**3,7%**
About once per week	39	**3,4%**
Several times per week	448	**38,9%**
Multiple times per day	603	**52,4%**
	**1151**	**100,0%**

How familiar are you with the concept of AI/ML in your daily life?		
Not at all familiar	56	**4,9%**
Slightly familiar (have heard about it, but minimal exposure)	159	**13,8%**
Somewhat familiar (have read about it, use it occasionally or seen demonstrations)	404	**35,0%**
Very familiar (routinely use AI tools e.g. ChatGPT, GEMINI, Siri)	535	**46,4%**
	**1154**	**100,0%**

How familiar are you with the concept of AI/ML in healthcare (not necessarily in POCUS)?		
Not at all familiar	78	**6,8%**
Slightly familiar (have heard about it, but minimal exposure)	265	**23,0%**
Somewhat familiar (have read about it or seen demonstrations)	633	**54,9%**
Very familiar (routinely use or study AI in some aspect of practice)	176	**15,3%**
	**1152**	**100,0%**

Have you ever used an AI- or ML-assisted tool in any area of patient care?		
Yes	260	**22,6%**
No	891	**77,4%**
	**1151**	**100,0%**

AI/ML-assisted POCUS could improve the speed of diagnosis in the ICU.		
Strongly disagree	6	**0,6%**
Disagree	20	**1,9%**
Neutral	254	**23,6%**
Agree	582	**54,2%**
Strongly agree	212	**19,7%**
	**1074**	**100,0%**

AI/ML-assisted POCUS would help improve the accuracy of my ultrasound interpretations.		
Strongly disagree	18	**1,7%**
Disagree	100	**9,3%**
Neutral	265	**24,7%**
Agree	495	**46,0%**
Strongly agree	197	**18,3%**
	**1075**	**100,0%**

Integrating AI/ML into my current POCUS workflow would be relatively seamless.		
Strongly disagree	17	**1,6%**
Disagree	134	**12,5%**
Neutral	271	**25,2%**
Agree	475	**44,2%**
Strongly agree	177	**16,5%**
	**1074**	**100,0%**

AI/ML-assisted POCUS could reduce inter-operator variability in ultrasound interpretation.		
Strongly disagree	5	**0,5%**
Disagree	18	**1,7%**
Neutral	97	**9,0%**
Agree	610	**56,7%**
Strongly agree	345	**32,1%**
	**1075**	**100,0%**

I would feel more confident in my clinical decisions if I had access to AI/ML-assisted interpretations.		
Strongly disagree	22	**2,0%**
Disagree	87	**8,1%**
Neutral	268	**24,9%**
Agree	453	**42,1%**
Strongly agree	245	**22,8%**
	**1075**	**100,0%**

I have sufficient training to use AI/ML-assisted ultrasound tools effectively.		
Strongly disagree	98	**9,3%**
Disagree	506	**48,1%**
Neutral	314	**29,8%**
Agree	102	**9,7%**
Strongly agree	33	**3,1%**
	**1053**	**100,0%**

Available training resources (e.g., workshops, online modules) are adequate to learn AI/ML-enhanced POCUS.		
Strongly disagree	96	**9,1%**
Disagree	473	**44,9%**
Neutral	305	**29,0%**
Agree	139	**13,2%**
Strongly agree	40	**3,8%**
	**1053**	**100,0%**

A lack of standardized training or credentialing for AI/ML in POCUS is a significant barrier.		
Strongly disagree	15	**1,4%**
Disagree	36	**3,4%**
Neutral	124	**11,8%**
Agree	471	**44,7%**
Strongly agree	407	**38,7%**
	**1053**	**100,0%**

Insufficient local expertise or technical support hinders the adoption of AI/ML-assisted POCUS.		
Strongly disagree	20	**1,9%**
Disagree	74	**7,0%**
Neutral	184	**17,5%**
Agree	470	**44,7%**
Strongly agree	304	**28,9%**
	**1052**	**100,0%**

I trust AI/ML algorithms to provide accurate ultrasound interpretations.		
Strongly disagree	3	**0,3%**
Disagree	60	**5,8%**
Neutral	252	**24,5%**
Agree	503	**48,8%**
Strongly agree	212	**20,6%**
	**1030**	**100,0%**

I am concerned that AI/ML errors could lead to incorrect diagnoses or treatments.		
Strongly disagree	5	**0,5%**
Disagree	62	**6,0%**
Neutral	406	**39,4%**
Agree	457	**44,4%**
Strongly agree	100	**9,7%**
	**1030**	**100,0%**

I would want to verify every AI/ML-generated finding with my own interpretation before making a clinical decision.		
Strongly disagree	3	**0,3%**
Disagree	53	**5,1%**
Neutral	214	**20,8%**
Agree	511	**49,6%**
Strongly agree	249	**24,2%**
	**1030**	**100,0%**

The 'black box' nature (lack of explainability) of AI/ML outputs reduces my trust in them.		
Strongly disagree	14	**1,4%**
Disagree	129	**12,5%**
Neutral	455	**44,2%**
Agree	347	**33,7%**
Strongly agree	85	**8,3%**
	**1030**	**100,0%**

Regulatory approval and strong evidence validating AI/ML tools would increase my willingness to use them		
Strongly disagree	2	**0,2%**
Disagree	10	**1,0%**
Neutral	128	**12,4%**
Agree	473	**45,9%**
Strongly agree	417	**40,5%**
	**1030**	**100,0%**

Implementing AI/ML in POCUS would slow down my workflow.		
Strongly disagree	59	**5,8%**
Disagree	441	**43,0%**
Neutral	365	**35,6%**
Agree	137	**13,4%**
Strongly agree	23	**2,2%**
	**1025**	**100,0%**

The cost of acquiring and maintaining AI/ML-enabled ultrasound machines is prohibitive.		
Strongly disagree	20	**2,0%**
Disagree	303	**29,6%**
Neutral	441	**43,0%**
Agree	173	**16,9%**
Strongly agree	88	**8,6%**
	**1025**	**100,0%**

Integration of AI/ML outputs into the health records (patient notes) is insufficient or too complex.		
Strongly disagree	25	**2,4%**
Disagree	386	**37,7%**
Neutral	423	**41,3%**
Agree	147	**14,3%**
Strongly agree	44	**4,3%**
	**1025**	**100,0%**

Additional technical support or staff would be required to effectively utilize AI/ML in POCUS.		
Strongly disagree	34	**3,3%**
Disagree	256	**25,0%**
Neutral	368	**35,9%**
Agree	299	**29,2%**
Strongly agree	68	**6,6%**
	**1025**	**100,0%**

Concerns about liability if AI/ML-assisted interpretations are incorrect discourage me from using these tools.		
Strongly disagree	34	**3,4%**
Disagree	282	**28,1%**
Neutral	399	**39,8%**
Agree	241	**24,0%**
Strongly agree	47	**4,7%**
	**1003**	**100,0%**

Data privacy and security concerns about patient information used to train AI systems are significant barriers		
Strongly disagree	100	**10,0%**
Disagree	410	**40,9%**
Neutral	291	**29,0%**
Agree	154	**15,4%**
Strongly agree	48	**4,8%**
	**1003**	**100,0%**

A lack of clear institutional or professional guidelines on AI/ML use in POCUS makes me hesitant to adopt it.		
Strongly disagree	14	**1,4%**
Disagree	59	**5,9%**
Neutral	123	**12,3%**
Agree	369	**36,8%**
Strongly agree	438	**43,7%**
	**1003**	**100,0%**

Cultural resistance to new technology among colleagues or leadership in my ICU inhibits the introduction of AI/ML tools.		
Strongly disagree	40	**4,0%**
Disagree	194	**17,0%**
Neutral	331	**33,0%**
Agree	319	**31,8%**
Strongly agree	120	**12,0%**
	**1004**	**97,7%**

Official endorsements or recommendations by professional societies would make me more willing to adopt AI/ML-assisted POCUS.		
Strongly disagree	33	**3,3%**
Disagree	104	**10,4%**
Neutral	276	**27,5%**
Agree	375	**37,4%**
Strongly agree	214	**21,4%**
	**1002**	**100,0%**

Section G: Overall Perceptions and Open-Ended Feedback Overall, I am enthusiastic about the potential role of AI/ML in improving POCUS practice.		
Strongly disagree	31	**3,1%**
Disagree	24	**2,4%**
Neutral	135	**13,4%**
Agree	542	**54,0%**
Strongly agree	272	**27,1%**
	**1004**	**100,0%**

In your opinion, what is the single greatest barrier to adopting AI/ML-assisted POCUS in your department?		
Training & Education	271	**27,1%**
Clinical Validation & Evidence	175	**17,5%**
Workflow Integration & Usability	66	**6,6%**
Trust & Transparency	74	**7,4%**
Cost & Accessibility	134	**13,4%**
Peer & Institutional Support	168	**16,8%**
Legal & Ethical Considerations	54	**5,4%**
Other	59	**5,9%**
	**1001**	**100,0%**
